# Regulation of Lipid Droplet Size in Mammary Epithelial Cells by Remodeling of Membrane Lipid Composition—A Potential Mechanism

**DOI:** 10.1371/journal.pone.0121645

**Published:** 2015-03-10

**Authors:** Bat-Chen Cohen, Avi Shamay, Nurit Argov-Argaman

**Affiliations:** 1 Department of Animal Sciences, The Robert H. Smith Faculty of Agriculture, Food and Environment, The Hebrew University of Jerusalem, Jerusalem, Israel; 2 Animal Science Department, The Volcani Center, The Ministry of Agriculture, Rehovot, Israel; Wageningen UR Livestock Research, NETHERLANDS

## Abstract

Milk fat globule size is determined by the size of its precursors—intracellular lipid droplets—and is tightly associated with its composition. We examined the relationship between phospholipid composition of mammary epithelial cells and the size of both intracellular and secreted milk fat globules. Primary culture of mammary epithelial cells was cultured in medium without free fatty acids (control) or with 0.1 mM free capric, palmitic or oleic acid for 24 h. The amount and composition of the cellular lipids and the size of the lipid droplets were determined in the cells and medium. Mitochondrial quantity and expression levels of genes associated with mitochondrial biogenesis and polar lipid composition were determined. Cells cultured with oleic and palmitic acids contained similar quantities of triglycerides, 3.1- and 3.8-fold higher than in controls, respectively (*P* < 0.0001). When cultured with oleic acid, 22% of the cells contained large lipid droplets (>3 μm) and phosphatidylethanolamine concentration was higher by 23 and 63% compared with that in the control and palmitic acid treatments, respectively (*P* < 0.0001). In the presence of palmitic acid, only 4% of the cells contained large lipid droplets and the membrane phosphatidylcholine concentration was 22% and 16% higher than that in the control and oleic acid treatments, respectively (*P* < 0.0001). In the oleic acid treatment, approximately 40% of the lipid droplets were larger than 5 μm whereas in that of the palmitic acid treatment, only 16% of the droplets were in this size range. Triglyceride secretion in the oleic acid treatment was 2- and 12-fold higher compared with that in the palmitic acid and control treatments, respectively. Results imply that membrane composition of bovine mammary epithelial cells plays a role in controlling intracellular and secreted lipid droplets size, and that this process is not associated with cellular triglyceride content.

## Introduction

Milk lipids are present in the form of milk fat globules (MFGs) that are secreted in a wide range of sizes, from 200 nm to over 15 μm, depending on the animal [1,2], genetic disposition [3], lactation stage [4], season [5] and diet [6,7]. The best established factor associated with MFG size is total fat content in the milk [8–10], suggesting a link between the metabolic and lipogenic capacity of mammary gland cells and MFG size. Nevertheless, the cellular mechanisms controlling MFG size are not clearly defined.

The size of MFGs is determined by their formation and secretion processes. MFG precursors, comprised of a triglyceride (Tg) core covered with a single layer of phospholipids, appear in the cytosol of mammary epithelial cells as microlipid droplets ranging in size from less than 0.5 μm to more than 4 μm [11,12]. The lipid droplets are transported to their secretion site, the apical pole of the cells. During the secretion process, the droplets can grow substantially in size upon fusion with other lipid droplets [13].

In adipose [14] and liver [15–17] cells, two main factors on the surface of the cytoplasmic lipid droplets—phospholipids and proteins—are suggested to control lipid droplet size. For example, in murine adipocytes, the level of perilipi-2, a member of the PAT-domain protein family, decreased concomitantly with a reduction in intracellular lipid droplet size upon dietary supplementation of trans-10, cis-12 conjugated linoleic acid [17]. In murine mammary gland cells, adipophilin knockout was associated with the formation of small intracellular lipid droplets relative to the wild type [18]. Other proteins with enzymatic activity are Tg-synthesis enzymes (GPAT, AGPAT and DGAT) and lipases, which are suggested to regulate lipid droplet size by increasing Tg synthesis or inducing Tg hydrolysis, respectively [19,20]. In addition, protein activity on the surface of the lipid droplet is suggested to be modulated by interactions with specific phospholipids, depending on the phospholipid head group [21]. In light of these findings, it is reasonable to assume that the composition of the phospholipid envelope plays a pivotal role in the regulation of lipid droplet size.

Indeed, phospholipid envelope composition has been shown to affect stability and consequently size of the lipid droplet in an aqueous cytoplasmic environment. Negatively curved, cone-like, phospholipids, such as phosphatidylethanolamine (PE), destabilize the lipid droplet membrane and hence induce lipid droplet fusion, resulting in larger droplets [19]. On the other hand, cylinder-like phospholipids, such as phosphatidylcholine (PC), have been suggested to stabilize the lipid droplet surface and hence inhibit fusion, resulting in smaller droplets in nematodes [22, 23] and *Drosophila* [24]. Whether these mechanisms are also involved in the modulation of lipid droplet size in the mammary epithelial cells has yet to be determined.

Because MFG size is associated with its phospholipid composition [3,7,25,26] it is likely that the composition of membrane surrounding the intracellular lipid droplet during its formation and secretion has a role in determining the MFG diameter.

The size of the MFG is tightly associated with its composition, with a higher content of mono- and polyunsaturated fatty acids and phospholipids in small vs. large MFGs [6,25,27]. A high content of mono- and polyunsaturated fatty acids in milk is of interest, due to their effects on human lipid metabolism. Moreover, a higher phospholipid content in milk may be desirable due to the recognized positive health effects related to plasma lipid profile and overall metabolism [28, 29], as well as beneficial effects on immune system, heart and brain functions [30]. MFG size also has industrial implications since it affects the physicochemical characteristics of dairy products as well as the aggregation and coalescence properties of milk [31–33]. These findings highlight the importance of understanding the mechanisms controlling MFG size from nutritional, human health and industrial perspectives. However, the possible involvement of membrane composition in the cellular mechanism controlling the size of both intracellular and secreted droplets is still illusive.

In the present study, phospholipid composition of mammary epithelial cells was studied in relation to intracellular and secreted lipid droplet structure. Data presented herein demonstrate that under conditions that induce Tg accumulation, lipid droplet size is regulated in a phospholipid composition-dependent manner.

## Materials and Methods

### Experimental design

Two sets of experiments were conducted. The first examined whether culturing with different free fatty acids (FFAs) induces changes in cellular Tg content and if so, whether these alterations are associated with membrane composition. The aim of the second experimental set was to test whether FFA-induced changes in membrane composition are associated with changes in the size of both intracellular and secreted droplets.

Three FFAs were used as treatments: capric, palmitic, and oleic acids, diluted with DMEM/F12 medium supplemented with 0.5% (w/v) FFA-free bovine serum albumin (BSA) to a final concentration of 0.1 mM. For the control, 0.5% FFA-free BSA was added to the medium. These fatty acids were chosen based on their affect on lipogenesis, lipid droplet formation and trafficking in adipose, liver and muscle [34–38].

Cells were plated at 200,000 cells per 60-mm plastic dish for cellular lipid extraction, or at 60,000 cells per 35-mm plastic dish on glass cover slips for Nile red or Mitotracker staining, as described further on. After overnight incubation, the medium was replaced with DMEM/F12 without serum, with 0.15% FFA-free BSA and insulin (1 μg/ml), hydrocortisone (0.5 μg/ml) and prolactin (1 μg/ml) for 48 h to induce milk lipid and protein synthesis. Then cells were treated with FFA for 24 h in the presence of insulin (1 μg/ml), hydrocortisone (0.5 μg/ml) and prolactin (1 μg/ml). After 24 h, cells were harvested for cell counting, lipid extraction, or staining of intracellular lipid droplets or mitochondria. In addition, after 24 h, medium was collected separately from each experimental group for Tg quantification and lipid droplet staining (see further on). A shorter incubation of 2 h with the FFA was performed before RNA extraction.

### Primary culture

#### Materials

DMEM/F12, penicillin, streptomycin, amphotericin B, L-glutamine solution, trypsin—EDTA solution C and fetal bovine serum (FBS) were all obtained from Biological Industries (Beit Haemek, Israel). Bovine insulin, hydrocortisone, ovine prolactin, BSA solution, hyaluronidase and DNase I were purchased from Sigma Aldrich Israel Ltd. (Rehovot, Israel). Collagenase type II was purchased from Worthington Biochemical Corporation (Lakewood, NJ).

#### Culture

Primary culture of mammary gland epithelial cells was isolated for mammary biopsies according to protocol established in our lab [39,40]. Briefly, udder tissue was collected of three lactating cows in a commercial slaughterhouse, and immediately submerged in DMEM/F12 medium supplemented with 10% (w/v) FBS, 100 U/ml penicillin, 100 μg/ml streptomycin, 0.25 μg/ml amphotericin B, 1 μg/ml insulin and 0.5 μg/ml hydrocortisone (growing medium). Study protocols were in compliance with the regulations of the Israeli Ministry of Health.

After transfer to the laboratory, 10 g of minced tissue was digested in 100 ml of growing medium supplemented with collagenase (1 mg/ml) and hyaluronidase (1 mg/ml) in a 500-ml Erlenmeyer flask shaken at 100 rpm for 3–4 h at 37°C. During tissue digestion, tissue fragments were mechanically dissociated by occasional aspiration through a 25-ml pipette with a large orifice. After incubation, the suspension was filtered through a Nitex mesh (250 μm) and the filtrate was centrifuged at 350*g* for 5 min. The supernatant was discarded and the cell sediment was treated with trypsin—EDTA solution C. The cells were then washed with the growing medium and treated with 0.04% (w/v) DNase, filtered through a 100-μm cell strainer (BD Falcon, Bedford, MA) and washed again with the growing medium. The medium was changed every 48 h. The cells were grown to confluence, dispersed with 0.05% (w/v) trypsin and transferred to new plates.

### Lipid extraction and analysis

#### Materials

For lipid extraction, analytical reagent-grade methanol and chloroform were purchased from Bio-Lab Ltd. Laboratories (Jerusalem, Israel). For HPLC analysis, HPLC-grade dichloromethane was purchased from Merck Millipore Mercury (Darmstadt, Germany), analytical reagent-grade methanol was purchased from Bio-Lab, and ammonium hydroxide solution (ca. 25% NH_3_) was purchased from Sigma (Rehovot, Israel). The Tg standard triolein (>99% pure) was purchased from Supelco (Bellefonte, PA). Cholesterol (<99% pure) and phospholipid standards were supplied by Sigma Aldrich, the latter consisting of PE (1,2-dioleoyl-sn-glycero-3-phosphoethanolamine, purity 99%), phosphatidyl inositol (PI) (L-α-phosphatidylinositol ammonium salt, from bovine liver, purity 98%), phosphatidylserine (PS) (1,2-dioleoyl-sn-glycerol-3-phospho-L-serine sodium salt, purity 95%), PC (1,2-dioleoyl-sn-glycero-3-phosphocholine, purity 99%), and sphingomyelin (SM) (from bovine brain, purity 97%).

#### Sample collection and lipid extraction

After the FFA-treated cells were harvested with trypsin, they were washed with 0.9% (w/v) NaCl and stored at -20°C until lipid extraction. Total lipids were extracted from the cells using a protocol adapted from the cold-extraction procedure developed by Folch et al. [41] and established in our laboratory [25]. A 5-ml aliquot of methanol:chloroform solution (2:1, v/v) was added to each sample. After incubation at room temperature, 1 ml of double-distilled water was added. After overnight incubation at 4°C, the upper phase was discarded and the lower phase was filtered through a Pasteur pipette with glass wool. Samples were then dried under a nitrogen stream at 65°C, diluted in 100 μl of chloroform:methanol (97:3, v/v) and stored at -20°C until injection for HPLC analysis.

#### Lipid concentration and composition

Separation of polar and neutral lipids was performed by HPLC (HP 1200, Agilent Technologies (Santa Clara, CA) combined with an evaporative light-scattering detector (1200 series ELSD, Agilent Technologies). The separation protocol consisted of a gradient of dichloromethane, 99% methanol and 1% ammonium, and double-distilled water (detailed in Table 1) using normal-phase chromatography on a silica column (Zorbax RX-SIL, 4.6 × 250 mm, Agilent Technologies). The column was heated to 40°C, flow was 1 ml/min, and injection volume was 20 μl. The ELSD was heated to 65°C, nitrogen pressure was 3.8 bar, the filter was set to 5, and gain (sensitivity) was set to 4 for the first 14 min, then changed to 12 until 21 min, and then changed to 7 until the end of the run to enable detection of differently abundant lipid components. The separation process was managed by ChemStation software (Agilent Technologies), which permitted the acquisition of data from the ELSD detector. Identification and quantification of the separated lipids were performed using external standards (Sigma Aldrich). Quantification was performed against external standard curves and expressed as amount per 10^6^ live cells. Live cell number was determined after Trypan blue staining, using a hemocytometer. Membrane composition is presented as weight percentage of each polar lipid out of the summed quantity of recognized phospholipids in each sample [25].

**Table 1:** HPLC Solvent Gradient Method Used for Quantifying Triglycerides, Cholesterol and Phospholipids Using Liquid Chromatography Equipped with ELSD Detector.

Time (min)	Dichloromethane (%)	Methanol* (%)	Water (%)
**0**	99	1	0
**8**	93	7	0
**12**	75	25	0
**16**	75	25	0
**20**	65	35	0
**22**	60	35	5
**33**	60	35	5
**36**	80	20	0
**39**	99	1	0
**44**	99	1	0

* The methanol included 1% ammonium hydroxide solution.

### Fluorescence staining

#### Intracellular lipid droplet staining

Cells grown on glass cover slips were rinsed three times with phosphate buffered saline (PBS) and fixed with 4% paraformaldehyde in PBS for 20 min at room temperature. Then the cover slips were rinsed four times with PBS and stained with Nile red (200 nM, Sigma) for 15 min. Cover slips were then rinsed three times with PBS and stained with DAPI (Sigma, St. Louis, MO) for 5 min. Then the cover slips were rinsed four times with PBS and mounted with fluorescence mounting medium (Dako, North America Inc., Carpinteria, CA).

Slides were visualized with an Olympus BX40 fluorescence microscope equipped with an Olympus DP73 digital camera using CellSens Entry software (version 1.7, Olympus), and with an Olympus IX81 confocal microscope using Fluoview 500 software. Lipid droplet diameter was measured using ImageJ software (version 1.48, NIH, Bethesda, MD). Cells with at least one lipid droplet larger than 3 μm were designated "large lipid droplets". Cells with droplets with a diameter of less than 3 μm were designated as "small lipid droplets". Cells with no visualized lipid droplet were designated as "no lipid droplets". In each field captured on camera, the mean diameter of the three largest lipid droplets was calculated and used to estimate the maximum diameter of the intracellular lipid droplet.

#### Staining of lipid droplets in the medium

After 24 h incubation with FFA, the medium was collected (5 ml) and centrifuged at 500*g* for 10 min. The supernatant was transferred to new tube and kept at 4°C until analysis. The medium samples were dried under a nitrogen stream at 37°C and diluted in 100 μl double-distilled water. Then, a 100-μl aliquot of 42 μg/ml Nile red solution was added, and samples were incubated for 2 h at room temperature. Then 5 μl of sample was deposited onto a glass slide and mounted with fluorescence mounting medium (Dako). Slides were visualized with an Olympus BX40 fluorescence microscope equipped with an Olympus DP73 digital camera using CellSens Entry software version 1.7. Lipid droplets were divided into three size groups: 0 > X < 3 μm, 3 ≥ X < 5 μm and X ≥ 5 μm). Data are presented as the percentage of lipid droplets in each size category out of the sum of all measured lipid droplets.

#### Determination of mitochondrion quantity

Cells were incubated in DMEM/F12 with 159 nM Mitotracker deep red FM stain (Cell Signaling Technology) for 30 min at 37°C. The cells were then fixed in ice-cold 100% methanol for 15 min at -20°C and rinsed three times with PBS. Cells were mounted with fluorescence mounting medium (Dako) and slides were visualized with an Olympus BX40 fluorescence microscope equipped with an Olympus DP73 digital camera using CellSens Entry software version 1.7. Mitochondrial fluorescence in each cell was quantified by ImageJ software version 1.48 using the following formula: Corrected total cell fluorescence = Integrated density of selected cell—(Area of selected cell x Mean fluorescence of background readings).

### Gene-expression analysis

#### RNA extraction and cDNA synthesis

Total RNA was isolated from primary mammary cells by EZ-RNA II total RNA isolation kit (Biological Industries) according to the manufacturer's instructions. The concentration and 260/280 nm optical density (OD) ratio of the RNA was determined by Nanodrop spectrophotometer (NanoDrop Technologies, Wilmington, DE). RNA samples were kept at -80°C until further analysis. Total RNA (1 μg) was reverse-transcribed to produce cDNA using the EZ first-strand cDNA isolation kit (Biological Industries) according to the manufacturer’s instructions.

#### Real-time PCR analysis

For gene-expression analysis, specific primers were designed by Primer-BLAST software (National Center of Biotechnology Information [NCBI], http://www.ncbi.nlm.nih.gov/tools/primer-blast/index) based on cDNA sequences published by the NCBI database or selected from the literature, as indicated (Table 2). Primers were synthesized by Sigma (Rehovot, Israel). cDNA was mixed with primers and platinum SYBR Green qPCR supermix—UDG without ROX (Invitrogen Corporation, Carlsbad, CA). An Mx3000P Real-Time PCR System (Stratagene, La Jolla, CA) was used with the following protocol: 2 min at 50°C, 2 min at 95°C, followed by 40 cycles of 30 s denaturation at 95°C, 1 min annealing and extension at 60°C. Analysis was performed by MxPro software version 4.10 (Stratagene). Dissociation-curve analysis was performed after each real-time experiment to confirm the presence of only one product and the absence of primer dimers. The efficiency of the reaction and the initial mRNA quantity in the sample was determined using DART-PCR software version 1.0. Expression data were normalized by geometrical means of two housekeeping genes: UXT and β2-microglobulin.

**Table 2:** Primer Sequences Used for Real-Time PCR Analysis.

**Gene**	Accession number	Sequence	Size (bp)	Reference
Keratin-18	NM_001192095.1	**F:** GGTCCCAGCAGATTGAGGAG **R:** CCCTCAGGCTGTTCTCCAAG	165	Self design
α-Lactalbumin	NM_174378	**F:** AAAGACGACCAGAACCCTCA **R:** GCTTTATGGGCCAACCAGTA	143	[53]
PGC-1α	NM_177945	**F:** GTACCAGCACGAAAGGCTCAA **R:** ATCACACGGCGCTCTTCAA	120	[46]
PGC-1β	XM_003586328.2	**F:** CACGGAGGAACTTCAGATGTGA **R:** GACAGGTTTCGAACGTACACCA	127	[46]
NDUFAF3	NM_001046105.2	**F:** ACGAGCTGTATCAACGGACG **R:** AACCTACGTTCCACTGCACC	162	Self design
PEMT	NM_182989.3	**F:** TTCTGGAATGTGGTTGCGAGA **R:** AGGACGTTCAAGAGCAGGATG	116	Self design
UXT	BQ676558	**F:** TGTGGCCCTTGGATATGGTT **R:** GGTTGTCGCTGAGCTCTGTG	101	[54]
β2-Microglobulin	NM_173893	**F:** CATCCAGCGTCCTCCAAAGAT **R:** CCCCATTCTTCAGCAAATCG	131	[46]

### Quantification of Tg in the culture medium

After 24 h incubation with FFA, medium was collected and centrifuged at 500*g* for 10 min. The supernatant was transferred to a fresh tube and kept at 4°C until analysis. Medium Tg quantification was performed with a triglyceride quantification kit (Abcam, Cambridge, UK) according to the manufacturer's instructions. The concentration of Tg in the medium was calculated per 10^6^ cells and presented as fold change relative to control.

### Statistical analysis

All statistical procedures were performed using JMP software version 7 (SAS Institute, Cary, NC). All reported data are means ± SEM. Treatment and experiment number were defined as fixed effects in the model. All dependent variables were checked for homogenic variance by unequal variances in JMP software and if the variance was not homogenic, a Welch-ANOVA test was performed. Comparisons were made by ANOVA followed by Tukey-Kramer HSD test.

The distribution of cell phenotypes based on lipid droplets size categories and size categories of lipid droplets in the medium was compared by chi-square test followed by Fisher's exact test. Significance probe was set to 0.05 and tendencies were reported at 0.05 < *P* ≤ 0.1.

## Results

### Culturing of mammary epithelial cells with FFAs changes the intracellular Tg content and ratio between polar and neutral lipids

**Fig 1:**
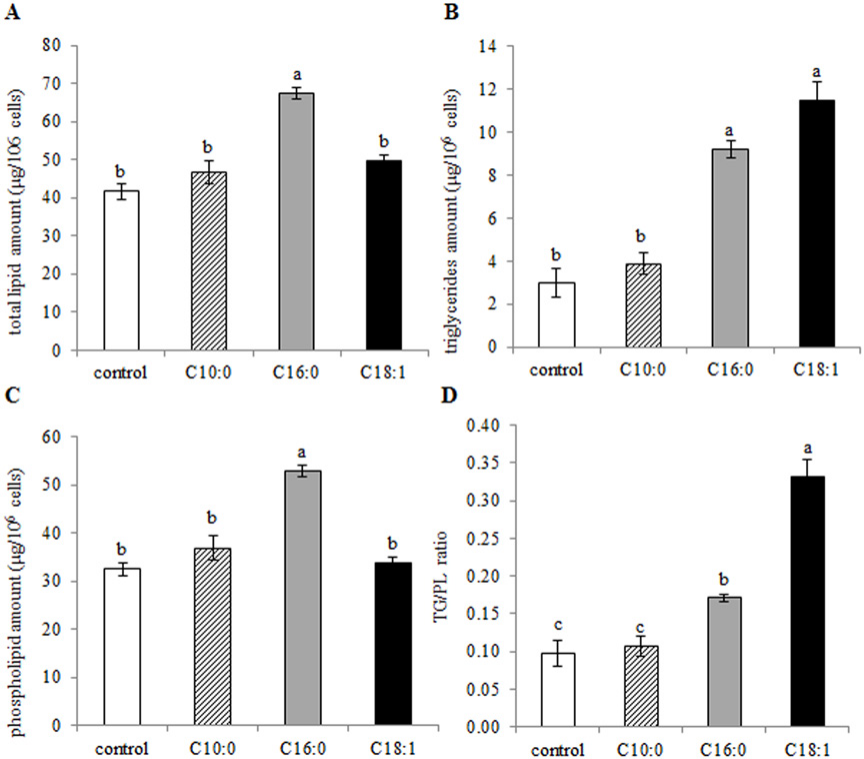
Specific free fatty acids (FFAs) influence amounts and ratio between polar and neutral lipids in mammary epithelial cells. Mammary epithelial cells were treated with 100 μM FFA (capric, palmitic or oleic acid) or with FFA-free medium (control) for 24 h; lipids were then extracted and analyzed by HPLC-ELSD. (A) Total lipid amount. (B) Triglyceride amount. (C) Total phospholipid amount. (D) Triglyceride-to-phospholipid ratio. All data are presented as mean ± SEM. Different letters indicate significant differences between treatment groups (*P* < 0.05).

In previous reports, specific fatty acids have been shown to induce, inhibit or have no effect on cellular lipogenic activity [34,35]. Therefore, primary culture of mammary epithelial cells was exposed to either capric, palimitic or oleic acid and total fat and Tg content, as well as the ratio between membrane and neutral lipids were determined. Total lipid amount in the cultured mammary epithelial cells was similar between control, capric and oleic acid treatments, whereas in cells cultured with palmitic acid, the total lipid amount was 62% higher than that in the control (*P* < 0.0001, Fig. 1A). The amount of Tg in the cells was 3.1- and 3.8-fold higher in the presence of palmitic and oleic acids, respectively, compared with the control (*P* < 0.0001, Fig. 1B), but did not differ between palmitic and oleic treatments. In addition, cellular Tg content did not differ between control and capric acid-treated cells. To evaluate the cellular lipid composition in terms of storage/secretion or membrane lipids, the ratio between Tg and total phospholipids was determined. Culturing mammary epithelial cells with capric acid did not change the Tg-to-total phospholipid ratio relative to the control. However, when cultured with palmitic and oleic acids, the ratio increased 1.7- and 3.4-fold, respectively, compared to the control (*P* < 0.0001, Fig. 1C). Moreover, the ratio between Tg and total phospholipids in the cells increased twofold in cells cultured with oleic acid vs. palmitic acid.

### Oleic and palmitic acids change membrane lipid composition

**Fig 2:**
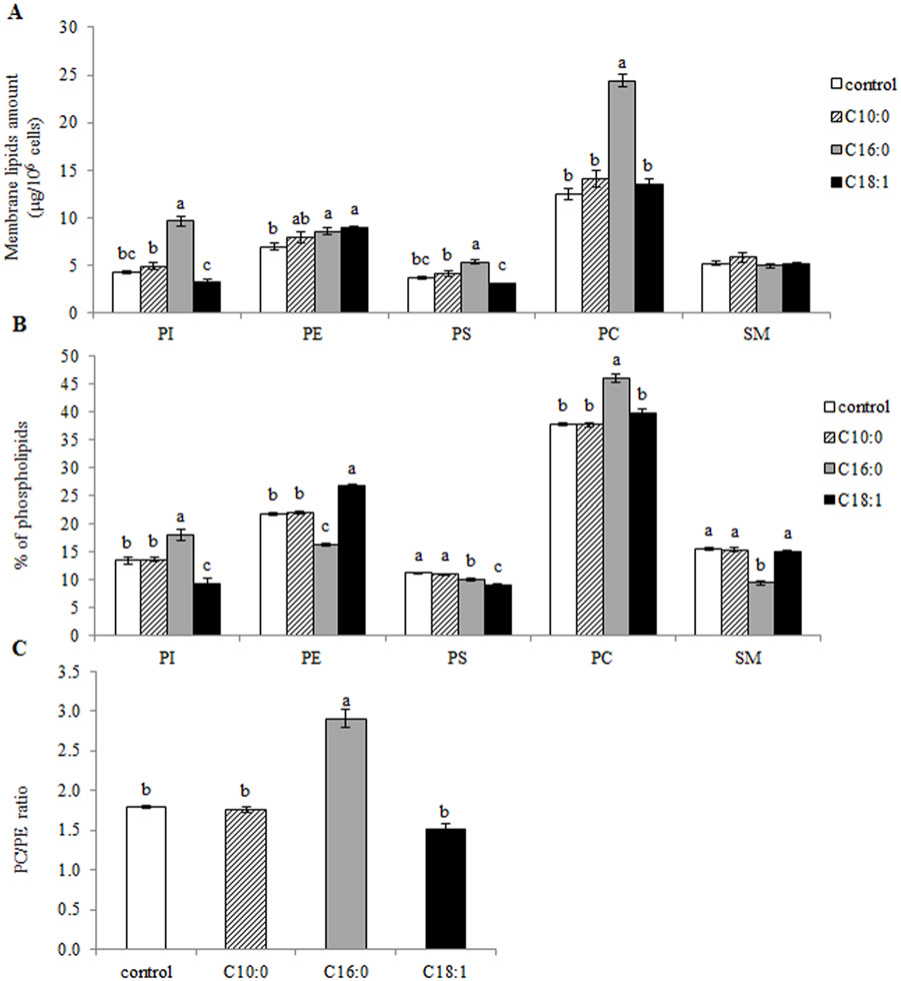
Specific free fatty acids (FFA) influence the amount and composition of phospholipid in mammary epithelial cells. Mammary epithelial cells were treated with 100 μM FFA (capric, palmitic or oleic acid) or with FFA-free medium (control) for 24 h; lipids were extracted and analyzed by HPLC-ELSD. (A) Membrane lipid amounts. (B) Phospholipid weight %. The percent of the amount of an individual phospholipid out of the summed phospholipids amounts. (C) Weight ratio between phosphatidylcholine and phosphatidylethanolamine. All data are presented as mean ± SEM. Different letters indicate significant differences between treatment groups (*P* < 0.05). PI: phosphatidylinositol; PE: phosphotidylethanolamine; PS: phosphatidylserine; PC: phosphotidylcholine; SM: sphingomyelin.

Lipogenic activity in an adipocyte cell line was found to be associated with changes in cellular phospholipid composition [33]. Therefore, we determined the amount and composition (weight %) of the main membrane phospholipids in mammary epithelial cells in the presence of different FFAs. Capric acid did not change phospholipid amounts compared to the control (Fig. 2A). The most prominent difference in polar lipid amount was found in the presence of palmitic acid, which increased PI, PC and PS by 2.2-, 2- and 1.5-fold, respectively, relative to the control (*P* < 0.0001). On the other hand, no differences were found in the cellular amounts of PI, PC and PS between oleic acid and control treatments. PE amount was higher by 23 and 29% in the presence of palmitic and oleic acids, respectively, compared to controls.

We then evaluated the effect of FFAs in the medium on membrane polar lipid composition calculated as the proportion of each polar lipid out of total phospholipids (weight %, Fig. 2B). In the presence of capric acid in the culture medium, no differences in membrane phospholipid composition were found. In the presence of palmitic acid, PI and PC were elevated by 34 and 22% whereas PE, PS and SM concentrations decreased by 25, 10 and 40%, respectively, compared to the control (*P* < 0.0001, Fig. 2B). In the presence of oleic acid, PE concentration was 23% higher whereas PI and PS decreased by 30 and 19%, respectively, compared to controls (*P* < 0.0001). The ratio between PE and PC has been suggested as a predictor for lipid droplet size [22]. In the current study, the ratio between PC and PE did not differ between control, capric and oleic acid treatments, whereas when cells were cultured with palmitic acid, the PE to PC ratio was 62% higher than that in the control (*P* < 0.0001, Fig. 2C).

### The influence of FFAs on the transcription levels of PGC-1 coactivators, a mitochondrial respiration enzyme and a phospholipid-converting enzyme

**Fig 3:**
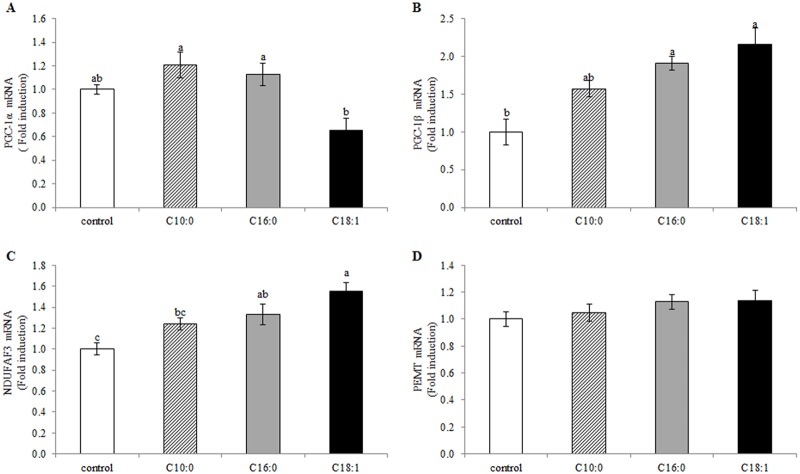
Transcription levels of activity markers of mitochondria and phospholipid converting enzyme are modulated by free fatty acids (FFAs). Mammary epithelial cells were treated with 100 μM FFA (capric, palmitic or oleic acid) or with FFA-free medium (control) for 2 h; RNA was extracted and gene-expression levels of (A) PGC-1α, (B) PGC-1β, (C) NDUFAF3 and (D) PEMT were analyzed by real-time PCR. All data are presented as mean ± SEM of the expression level of the assayed gene normalized to the geometric mean of two housekeeping genes. Different letters indicate significant differences between treatment groups (*P* < 0.05).

PGC-1 coactivators have been shown to regulate mitochondrial biogenesis [34,35]. Therefore, in the present study, their gene-expression levels were determined as an indicator of mitochondrial quantity (Fig. 3). PGC-1α transcription levels were approximately 50% lower in the oleic acid treatment than in the palmitic and capric acid treatments (*P* = 0.001, Fig. 3A). PGC-1ß was elevated by both palmitic and oleic acid treatments, by 91 and 116%, respectively (*P* = 0.0001, Fig. 3B) compared to the control. Capric acid did not change PGC-1α or ß transcription levels compared to the control.

NDUFAF3 has a role in mitochondrial oxidative phosphorylation, and its transcription levels increase in the initial stages of lactation in the mouse mammary gland [42]. Its transcription levels were therefore chosen as a measure of mitochondrial number in the present study (Fig. 3C). Both palmitic and oleic acids increased NDUFAF3 transcription levels by 33 and 55%, respectively, relative to the control (*P* = 0.0005). NDUFAF3 levels did not differ between the capric acid treatment and the control.

One of the pathways for PC synthesis is sequential methylation of PE, facilitated by phosphatidylethanolamine methyl transferase (PEMT) [43]. In the present study, PEMT transcription levels were similar in all treatments (*P* = 0.37, Fig. 3D).

### Fluorescence staining

Fluorescence staining was performed on control, palmitic acid and oleic acid treatments to determine the effect of increased Tg concentration in the cells and altered membrane composition on mitochondrial quantity and on the size of intracellular and secreted lipid droplets.

#### Oleic acid increases mitochondrial quantity in the mammary epithelia

**Fig 4:**
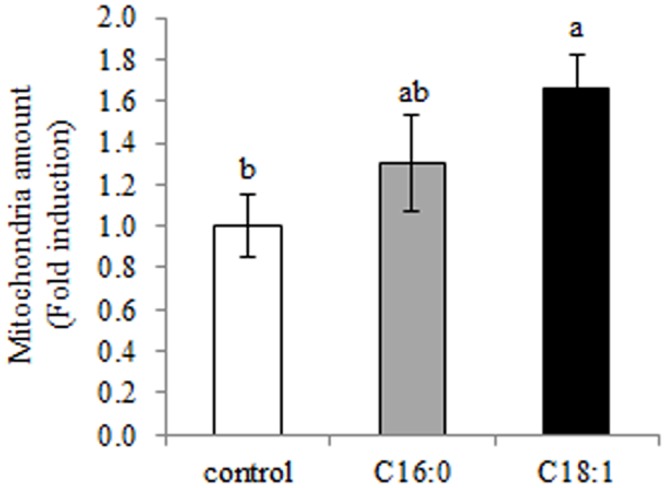
Specific free fatty acids (FFAs) in culture medium alter mitochondrial quantity in mammary epithelial cells. Mammary epithelial cells were treated with 100 μM FFA (palmitic or oleic acid) or with FFA-free medium (control) for 24 h, and mitochondria were stained with Mitotracker deep red FM. Mitochondrial amount was determined by quantification of the fluorescence intensity of Mitotracker deep red staining and presented as fold change compared to the control. Data are presented as mean ± SEM. Different letters indicate significant differences between treatment groups (*P* < 0.05).

Mitochondria play a role in lipogenesis and phospholipid conversion. Therefore, mitochondrial quantity was determined in controls and in the presence of palmitic and oleic acids in the culture medium (Fig. 4). In the presence of oleic acid, a 66% increase in mitochondrial quantity was found relative to the control (*P* = 0.027). Although a numerical increase of 30% in mitochondrial quantity was found in the presence of palmitic acid over controls, the increase was not significant. It should be noted that Mitotracer stains all of the mitochondria regardless of their functionality level and therefore can only be used to quantify them.

#### Oleic and palmitic acids change the distribution of cell phenotypes categorized by lipid droplet size and lipid droplet maximal diameter

**Fig 5:**
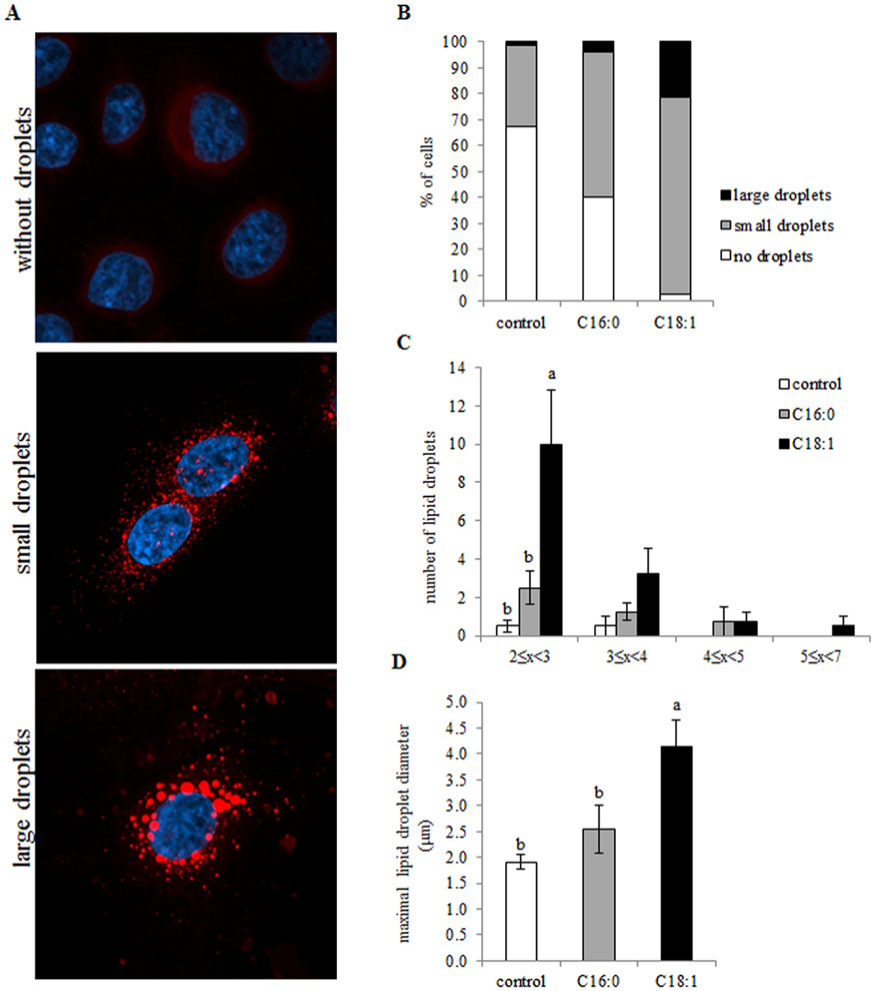
Intracellular lipid droplet size is altered by the presence of various free fatty acids (FFAs) in the culture medium. After cultivating mammary epithelial cells with 100 μM FFA (palmitic or oleic acid) or with FFA-free medium (control) for 24 h, lipid droplets were stained with Nile red. (A) Representative images showing the cellular phenotype according to the presence and size of cytoplasmic lipid droplets. Cells were categorized into three groups according to their lipid droplet phenotype: without lipid droplets, with small lipid droplets, or with large lipid droplets. Scale bar = 10 μm. (B) Distribution of mammary epithelial cells with different lipid droplet phenotypes was analyzed by chi-square test (*P*> 0.05). (C) Number of lipid droplets, by size categories. (D) Maximal lipid droplet diameter. In C and D, data are presented as mean ± SEM and different letters indicate significant differences between treatment groups (*P* < 0.05).

Phospholipid composition has been found to be associated with lipid droplet size in *Drosophila* S2 cells [24], *Caenorhabditis elegans* [22] and an adipocyte cell line [14]. Therefore, mammary epithelial cells in the present study were stained to visualize intracellular lipid droplets and to determine their size. When visualized under a fluorescence microscope, cell heterogeneity was observed in all treatments with respect to presence and diameter of lipid droplets. Therefore, cells were split into three categories according to their lipid droplet phenotype (Fig. 5A), and then the pattern of cell distribution according to lipid droplet size was determined (Fig. 5B). Different patterns were found among the three treatments (*P* < 0.0001). Cells with large lipid droplets were found almost exclusively in the oleic acid treatment. In the presence of oleic acid, 22% of the cells were categorized as containing large droplets compared with only 2% in the control and 4% in the palmitic acid treatment. In the control, 67% of the cells were categorized as having no lipid droplets, while only 2.5% of the cells in the oleic acid treatment were in this category. Most of the cells in both palmitic and oleic acid treatments, but only 31% of the cells in the control, contained small lipid droplets.

Next, the number of lipid droplets in a specific diameter range was determined for each treatment (Fig. 5C). The largest lipid droplets, ranging from 5 to 7 μm, were found only in the presence of oleic acid in the culture medium. A similar number of 4- to 5-μm diameter lipid droplets were found in the palmitic and oleic acid treatments, but not in the control. The number of 3- to 4-μm diameter lipid droplets was twofold higher in the oleic acid vs. palmitic acid treatments and in the palmitic acid vs. control treatments, but due to the large variations between cells, this difference was not significant (*P* = 0.12). Lipid droplets ranging from 2 to 3 μm in diameter were found in the three treatments, with the lowest number in the control and the highest number in the oleic acid treatment (*P* = 0.04). The number of these lipid droplets was twofold higher in the palmitic acid treatment than in the control, and fivefold higher in the oleic acid vs. palmitic acid treatment.

To quantify the maximal diameter of intracellular lipid droplet, the diameters of the three largest droplets in the cells were determined per treatment. In the presence of oleic acid, the average maximal diameter was 2-fold that in the control and 1.6-fold that in the palmitic acid treatment (Fig. 5D, *P* = 0.0139).

#### Size of lipid droplets secreted into the culture medium

**Fig 6:**
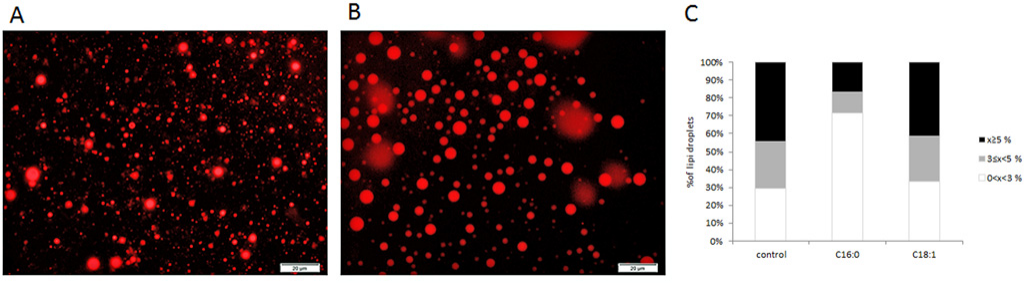
Different size distribution of lipid droplets in the medium induced by specific free fatty acids (FFAs). After cultivating mammary epithelial cells with 100 μM FFA (palmitic or oleic acid) or with FFA-free medium (control) for 24 h, medium was collected and lipid droplets were stained with Nile red. Representative images of lipid droplets in the medium collected from palmitate and oleate treatments (A and B, respectively). Droplets were measured and divided into three size groups: 0 > X < 3, 3 > X < 5, and ≤5 μm. Size distribution of lipid droplets in the medium was compared by chi-square test (C). Scale bar = 20 μm.

Lipid droplets in the medium were stained and their size patterns determined (Fig. 6). Different patterns were found in the control, oleic acid and palmitic acid treatments (*P* < 0.0001). The size distribution was similar between oleic acid and the control but differed in the palmitic acid treatment. In control and oleic acid treatments, 44 and 41% of the lipid droplets were larger than 5 μm, whereas in the palmitic acid treatment only 16% of the droplets were in this size category. In the palmitic acid treatment, the diameters of over 70% of the lipid droplets ranged between 0 and 3 μm, whereas in the control and oleic acid treatments, these droplets accounted for only 33 and 29% of all measured droplets, respectively.

### Tg content in the culture medium

**Fig 7:**
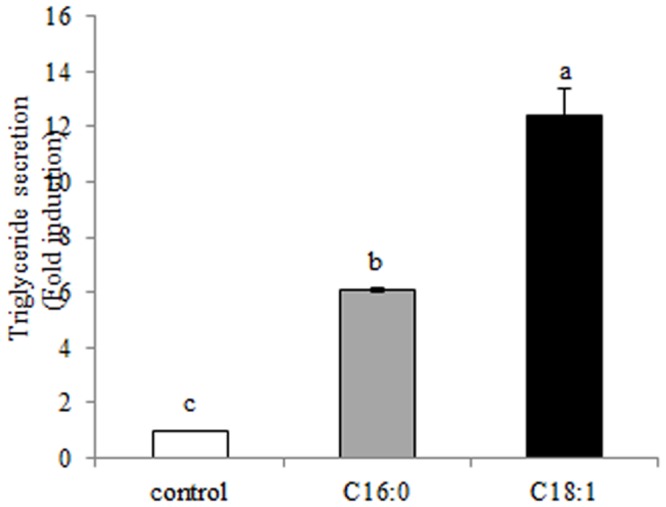
Triglyceride (Tg) secretion from mammary epithelial cells is altered in the presence of specific free fatty acids (FFAs). Mammary epithelial cells were treated with 100 μM FFA (palmitic or oleic acid) or with FFA-free medium (control) for 24 h, then the medium was collected and Tg content was determined. Data are presented as mean ± SEM. Different letters indicate significant differences between treatment groups (*P* < 0.05).

To evaluate the lipid-secretion capacity of the mammary epithelial cells under the various treatments, the concentration of Tg in the culture medium was determined (Fig. 7). Tg secretion was 12-fold and 2-fold higher in the presence of oleic acid vs. the control and palmitic acid treatments, respectively (*P* = 0.0003).

## Discussion

In the current study we provide first evidence for a link between intracellular and secreted lipid droplet size and membrane composition in mammary gland epithelial cells. One of the most remarkable results was that lipid droplet size and secretion rates were modified concomitantly with cellular phospholipid composition, regardless of the amount of Tg in the cell.

Exposing mammary gland epithelial cells to FFAs changed the ratio between Tg and membrane phospholipids (Fig. 1). Although both palmitic and oleic acids increased Tg content in the cells to the same extent, the Tg-to-total phospholipid ratio was much higher in the oleic acid treatment (Fig. 1). This implies that oleic acid was incorporated into Tg to a much higher extent than palmitic acid, as has been reported for Chinese hamster ovary cells [36]. Of all of the measured phospholipids, the greatest impact of palmitic acid was on PC amount, which suggests preferred incorporation of palmitic acid into PC. This finding is in agreement with the fact that in the mammary gland, palmitic acid content is highest in PC compared to all other phospholipids [44].

Different FFAs also considerably changed the cellular amounts of PS and PE (Fig. 2A). In light of this, we assume that the higher PE and lower PS amounts in the presence of oleic acid result from conversion of phospholipids through an enzymatic chain reaction in which PC is converted to PS by phoshatidylserine synthase, and then further converted to PE by phosphatidylserine decarboxylase. Finally, PE can be converted back to PC by PEMT [43]. The first conversion step of PS to PE is facilitated by phosphatidylserine decarboxylase which is located in the mitochondria [43]. Therefore, it is reasonable that the higher mitochondrial quantity found in the current study in the oleic acid treatment (Figs. 3 and 4) increases the conversion of PS to PE, resulting in higher PE content. In addition, the gradual elevation in mitochondrial quantity in the palmitic acid and oleic acid treatments corresponds to the gradual increase in transcription levels of PGC-1ß (Fig. 3). Both PGC-1ß and α have been shown to increase mitochondrial biogenesis in liver and muscle cells [37, 38, 45] in response to FFAs. Therefore, one might expect the same transcription pattern for both PGC-1 proteins. However, our findings revealed an increased in PGC-1ß levels in the palmitic acid and oleic acid treatments, whereas that of PGC-1α was reduced by oleic acid or remained unchanged with palmitic acid, relative to the control. Different transcription patterns of the two PGC-1 coactivators have also been reported in in-vivo and in-vitro studies in mammary gland cells [46,47].

In the palmitic acid treatment, PE amounts were higher than in the control, but no reduction in PS amounts was noted (Fig. 2A). On the contrary, PS amounts under the palmitic acid treatment were higher than in the control. We hypothesize that the palmitic acid-increased PE is attributed to the increased amount of PC, as the phospholipid conversion chain utilizes PC as a substrate, converting it to PS and then to PE. In addition, no differences were found between treatments in PEMT transcription levels (Fig. 3), suggesting that PC synthesis through the PEMT pathway is only secondary in this system, as previously demonstrated for liver cells [48,49]. Taken together, these results suggest that the elevation in PE amounts in the presence of palmitic and oleic acids is facilitated through different pathways.

The changes in phospholipid amounts were also reflected in the differential phospholipid compositions in the cells exposed to the different FFA treatments (Fig. 2B). Moreover, different membrane phospholipid composition corresponded with different intracellular lipid droplet size: over 20% of the cells exposed to oleic acid contained large droplets, whereas in the palmitic acid treatment, almost none of the cells were included in the "large lipid droplet" category (Fig. 5B). In addition, oleic acid increased the intracellular lipid droplet diameter approximately twofold compared to the palmitic acid treatment (Fig. 5D). The smaller intracellular lipid droplets found in the presence of palmitic acid were associated with higher PC concentrations in the cellular membrane. PC stabilizes the lipid droplet surface, thereby inhibiting droplet fusion and resulting in most of the cells in this treatment having small lipid droplets (Fig. 5B). A negative association between PC content and lipid droplet size has also been found in *Drosophila* S2 cells [24], nematodes [23] and mouse fatty livers [50]. On the other hand, when cultured with oleic acid, intracellular PC concentration was similar to that of the control, but PE concentration was higher. Higher PE content might induce droplet fusion, resulting in larger droplets in the cell cytoplasm [14], as found in the current study for the oleic acid treatment. As palmitic and oleic acid treatments were indistinguishable in terms of intracellular Tg amounts, the intracellular lipid droplet size is suggested to be regulated independently of cellular Tg content. This has also been demonstrated in the liver of *fa/fa* Zucker rats exposed to different isomers of conjugated linoleic acid [17]. Hence these results imply that membrane composition is the driving force for the bioproduction of specific sizes of lipid droplets.

It has been previously suggested that the ratio between PC and PE determines the stability of the lipid droplet and hence its size. In the present study, small intracellular lipid droplets found in the palmitic acid treatment were associated with a higher PC-to-PE ratio (Fig. 2C). A similar change in PC-to-PE ratio and droplet size was found in differentiating preadipocytes [14]. However, in nematodes, a higher PC-to-PE ratio was found in large vs. small droplets [22]. This inconsistency might be attributed to the fact that the latter study design included depletion of steroyl CoA desaturase activity which is essential for lipid droplet expansion.

In the current study, the intracellular PC-to-PE ratio was also associated with the size of the lipid droplets secreted into the medium: the size of droplets secreted from cells cultured with palmitic acid were characterized by a high proportion of small droplets; on the other hand, both oleic acid and control treatments resulted in the secretion of large lipid droplets in the culture medium and were indistinguishable in their intracellular PC-to-PE ratio (Fig. 6). Taken together, this provides first evidence for the relevance of the PC-to-PE ratio to the size of secreted lipid droplets, which up until now had only been shown in relation to the size of intracellular lipid droplets.

The larger droplets in the oleic acid treatment were associated with higher Tg concentration in the medium as compared to the palmitic acid treatment (Fig. 7). The greater Tg secretion in the presence of oleic acid might be attributed to the higher PE content, which destabilizes the membrane and increases its curvature [19, 51], thereby potentially inducing secretion. The importance of PE in the secretion process has been demonstrated in *Escherichia coli*, where depletion of PE from the cells nearly abolished its secretion capacity [52].

Taking the medium collected in this experiment as a compositional equivalent to milk implies that the biochemical processes leading to modulated membrane composition will determine milk lipid content, composition and structure, regardless of the net amount of Tg in the cells.

## Conclusion

**Fig 8:**
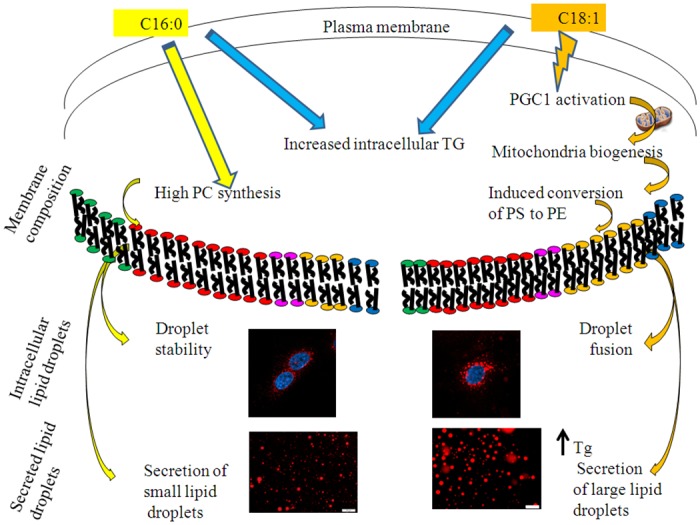
Suggested mechanism for free fatty acids effect on intracellular and secreted lipid droplets in mammary gland epithelial cells. The presence of free palmitic or oleic acid in the culture medium increased Tg amount in mammary epithelial cells. In addition oleic acid induced changes in the PGC-1 coactivators transcription pattern which increased mitochondria number. Consequently, higher conversion rates of PS to PE occurred and resulted in altered membrane composition. The higher PE content in the membrane induced fusion of intracelllular lipid droplets which results in larger lipid droplets in the cytoplasm. The higher PE also increased plasma membrane curvature and hence increased the secretion rates of the large lipid droplets which resulted in higher Tg concentration in cultrue medium after incubation with oleic acid. In the presence of palmitic acid (left hand side of the illustration) in the culture medium Tg content was also elevated and also proffered incorporation of palmitic acid into PC increased its content in the cellular membranes. PC induced membrane stability which inhibited the fusion rates of intracellular lipid droplets and resulted in small intracelluar and secreted lipid droplets. This suggested mechanism may explain the reason for the association between the size of the milk fat globules and the membrane phospholipid composition of mammary epithelial cells. C16:0- palmitic acid. C18:1- oleic acid. PGC1- PPAR gamma coactivator 1.PS- Phosphatidylserine. PE- Phosphatidylethanolamine. PC- Phosphatidylcholine. Tg- Triglyceride. Orange arrows- pathways induced by oleate. Yellow arrows- pathways induced by palmitate. Blue arrows- pathways induced by both oleate and palmitate. Membrane composition: Green- phoaphatidylinositol. Red- Phosphatidylchole. Pink- Phosphatidylserine. Yellow- Phosphatidylethanolamine. Blue- Sphingomyelin.

The data from this study suggest that membrane phospholipid composition plays a role in regulating MFG size. The association of a specific membrane composition with lipid droplet phenotype provides novel insight into the biochemical mechanism underlying the regulation of MFG size. The results enhance our understanding of how MFG size can be regulated and consequently, how lipid composition of milk can be determined and the suggested mechanism is illustrated in Fig. 8. Data presented herein may provide a novel means of controlling, and ultimately improving milk lipid composition.
